# Two-dimensional shear wave elastography of liver in healthy dogs: anaesthesia as a source of variability

**DOI:** 10.1080/23144599.2022.2073138

**Published:** 2022-05-23

**Authors:** Caterina Puccinelli, Tina Pelligra, Angela Briganti, Simonetta Citi

**Affiliations:** Department of Veterinary Sciences, University of Pisa, Pisa, Italy

**Keywords:** Shear wave elastography, liver, anaesthesia, dog

## Abstract

Two-dimensional shear wave elastography (2D-SWE) is a non-invasive method to quantitatively evaluate the liver stiffness (LS), allowing the detection of hepatic pathological changes in both dogs and humans. In dogs, some factors such as patient movement and respiration can cause artefacts and potential errors of measurements. Therefore, anaesthesia has been suggested to reduce the effect of the movement on 2D-SWE in dogs. This study was performed to evaluate the influence of an anaesthetic protocol on 2D-SWE measurements for assessment of LS in healthy dogs. Forty-five dogs were included and subjected to anaesthesia: in 11 dogs, the 2D-SWE was performed both before and under anaesthesia, in 19 dogs, the 2D-SWE was performed only when they were awake and in 15 dogs, the examination was carried out only under anaesthesia. The anaesthetic protocol was composed of intramuscular injection of a combination of dexmedetomidine, methadone and ketamine and intravenous administration of propofol for induction and isoflurane for maintenance. The variability of 2D-SWE values according to anaesthesia was evaluated. Median 2D-SWE values were significantly higher in anesthetized dogs compared to awake dogs either by considering separately the dogs in which the examination was performed both awake and under anaesthesia and by considering all dogs included. According to our study, anaesthesia helped to avoid challenges related to patient movement and respiration; however, it was a source of variability on 2D-SWE values, and this factor should be considered before performing 2D-SWE under anaesthesia.

## 1. Introduction

Shear wave elastography (SWE) is a non-invasive ultrasonographic method, which allows the evaluation of parenchymal rigidity, measuring the speed of the shear waves in tissues, which, being linked with stiffness, can be converted into kilopascals (kPa), the unit of Young’s modulus [1,2]. Shear wave elastography comprehends vibration-controlled transient elastography (VCTE) and acoustic radiation force impulse (ARFI) techniques[1]. In ARFI techniques, among which the two-dimensional shear wave elastography (2D-SWE), the assessment of tissues biomechanical properties is performed by applying an internal push pulse of a focused ultrasound beam that generates a tissue deformation and the creation of shear waves[1].

In human medicine, the liver is an important target organ for the use of SWE, whose main application is to assess the level of fibrosis in chronic liver diseases, to support the evaluation of prognosis and to guide the management[2]. In veterinary medicine, few studies have been performed in dogs about the use of SWE for the assessment of liver stiffness (LS) and they showed SWE to be able to detect pathologic changes in this species, too [3–8]. Some studies carried out in dogs have shown that liver values of SWE could be higher in course of specific pathologic conditions, such as hepatic fibrosis, extrahepatic biliary obstruction and brachycephalic obstructive air syndrome, compared to those of healthy patients [5,6,8]. However, further research is warranted to evaluate the feasibility and utility of this technique for the study of liver parenchyma in dogs [5,7].

Several causes of possible failure or alteration in the acquisition of SWE measurements have been described in humans, including deep inspiration and poor breath hold [2,9–11]. In human medicine, a transient breath hold in a neutral position, avoiding deep inspiration, is requested during the acquisition of the measurements[2]. In dogs too, movement and respiration could represent a possible source of failure or difficulty in performing SWE[3]. Therefore, anaesthesia has been suggested to reduce the motion effect on SWE in dogs, allowing the examination also in uncooperative patients when necessary[3]. Some technical factors that could affect SWE values of the liver of dogs, such as different measuring sites, depth from the liver capsule and different approaches, have been evaluated [3,7], but the possible influence of anaesthesia has not been studied yet.

The primary aim of our study was to evaluate the influence of one anaesthetic protocol on 2D-SWE measurements for assessment of LS in a group of healthy dogs; the secondary aim was to evaluate the incidence of the need of anaesthesia to perform a correct SWE study in a group of animals. Our hypothesis is that anaesthesia could interfere with the SWE values, but that it could be mandatory in a certain number of animals in order to correctly perform the examination.

## 2. Material and methods

The investigation was prospective. Case inclusion was performed at the Veterinary Teaching Hospital of the Department of Veterinary Sciences, University of Pisa.

### 2.1. Study population

Dogs included were admitted to perform an orthopaedic radiographic examination under anaesthesia for the preventive or certificate study for elbow and hip dysplasia or for pre-operative planning for cranial cruciate ligament rupture. All the dogs were considered healthy based on history, complete blood count, serum biochemical analysis and abdominal ultrasonography.

For the anaesthetic procedure and 2D-SWE, dogs were fasted for 12 hours, and a client consent was obtained to enrol the dogs in the study.

### 2.2. 2D-SWE measurement

Two 2D-SWE measurements were obtained: the first 2D-SWE was performed before the anaesthesia, and once the examination was completed, the anaesthetic protocol was started. Regarding the procedures carried out during the anaesthesia, the radiographic examination was carried out first, and then the second 2D-SWE was performed. The time elapsed between the premedication and the second 2D-SWE had to be between 30 and 60 minutes.

**Figure 1.**
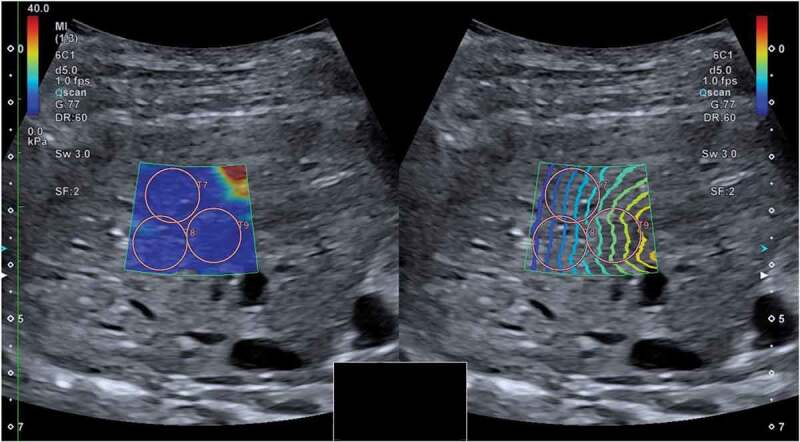
Two-dimensional shear wave elastography (speed mode on the left and propagation mode on the right) of one dog included in the study, performed when the dog was awake. Shear wave velocities were measured in the parenchyma of the right liver lobe. The speed mode displays a colour gradient with increasing shear wave velocity represented by an ascending order of blue, yellow and red. The propagation mode displays the propagating shear wave within tissue as contour lines. Three regions of interest (T7, T8 and T9) have been positioned inside the sample box.

Because it was not possible to perform the 2D-SWE before and under anaesthesia in all dogs included, they were retrospectively divided into three groups, considering when the examination was performed (awake and/or under anaesthesia), as follows group 1, before and during anaesthesia; group 2, only awake; and group 3, only under anaesthesia. (Figure 1)

The 2D-SWE was performed using a Canon Aplio a CUS-AA000 (Canon Medical Systems Europe B.V., Zoetermeer, the Netherlands), with a 3.5 MHz convex probe (PVT-375 BT) by one sonographer (T.P.).

The 2D-SWE was carried out following the technique previously described in dogs in the studies by Tamura et al. [4,5,8], according to the human liver elastography guidelines [1,2].

All dogs were positioned in the left lateral recumbency to allow the imaging of the right lobe of the liver. An intercostal approach was used, and the probe was placed parallel to and within one of the last right intercostal spaces to visualize the right lobe of the liver, after the clipping of the correspondent skin area and an application of a sufficient amount of gel. The 2D-SWE was performed during the end-expiratory phase of respiration to minimize effects of respiratory motion.

The ultrasound system during the acquisition of the images showed the sample box in two display modes at the same time: the speed mode and the propagation mode. The speed mode displayed a map of the shear wave velocity, and the propagation mode showed the propagation of the shear wave within tissues as contour lines. Parallel counter lines should be used to guide the placement of the region of interest (ROI), to obtain an accurate measurement.

To avoid reverberation artefacts from the liver capsule, the sample box was placed at least 10 mm beneath the liver capsule. The ROIs were positioned inside the sample box within a depth of 45 mm. The diameter of the ROIs was set to 10 mm. Each ROI was placed in an area of the hepatic parenchyma showing parallel lines in the propagation mode, excluding major vessels. (Figure 1) At least 10 valid measurements were obtained for each dog. Median values of shear wave velocity expressed in metre per second (m/s) were calculated and also converted in kilopascal (kPa). We used an interquartile range to the median ratio (IQR/M) ≤ 30%, according to the liver elastography guidelines originally developed from TE in human medicine [1,2].

### 2.3. Anaesthetic protocol

Dogs were sedated with a combination of dexmedetomidine (5 mcg/kg), methadone (0.2 mcg/kg) and ketamine (1 mg/kg) IM in the same syringe, then induced with propofol IV to effect to obtain endotracheal intubation and maintained with isoflurane.

### 2.4. Statistical analysis

Statistical analyses were performed using a commercially available statistical software package (GraphPad Prism, GraphPad Software Inc, San Diego, CA, USA). Descriptive statistics were generated. Data were assessed for normality using the Shapiro-Wilk test and expressed as the median and range. The variability of 2D-SWE values according to anaesthesia was evaluated with the Wilcoxon matched-pairs signed-rank test and the Mann Whitney test, respectively, for comparison of paired (group 1) and unpaired samples (group 1 + group 2 vs group 1 + group 3).

### 2.5 Ethical statement

The research protocol was approved by the Institutional Animal Care and Use Committee of the University of Pisa (permission number:43/2020).

## 3. Results

Forty-five dogs, undergoing diagnostic orthopaedic procedures under anaesthesia, were included.

The dogs included were 24 females and 21 males, with a median age of 1.9 years (range, 0.5–11 years) and a median body weight of 27.8 kg (range, 4–40 kg). The breeds represented in the study were Labrador Retriever (n = 20), Golden Retriever (n = 9), mixed breed (n = 7), Jack Russell Terrier (n = 2), English Setter (n = 2) and one for each of the following breeds: American Pitbull Terrier, Poodle, Border Collie, Schnauzer and Gordon Setter.

In 11 (24.4%) dogs, the 2D-SWE was performed before and during anaesthesia (group 1). In 19 (42.2%) dogs, the 2D-SWE was performed only when they were awake (group 2), due to organizational reasons or to not exceed anaesthesia time, and in 15 (33.3%) dogs, only during anaesthesia (group 3), because it was not possible to carry out the examination when they were awake due to movement of the patient and/or tachypnoea.

For anaesthetized dogs, the time elapsed between the premedication and the 2D-SWE was about 40–45 minutes.

**Figure 2.**
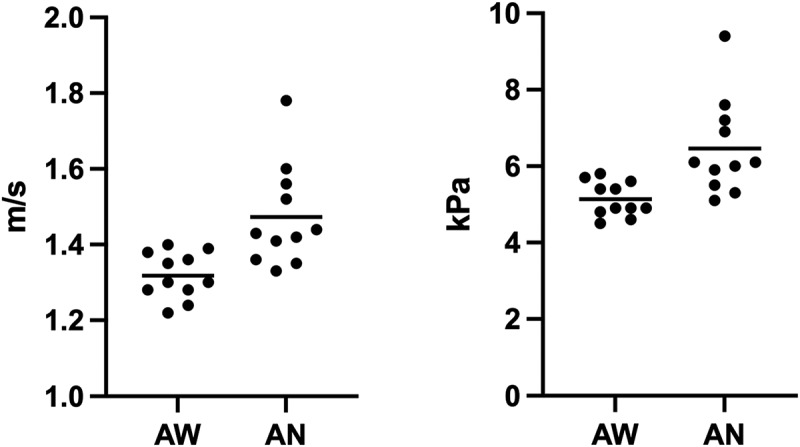
Scatter plots of the shear velocity values in awake dogs (AW) and anaesthetized dogs (AN) for group 1, expressed in metre per second (m/s) and kilopascal (kPa).

The median 2D-SWE values of group 1 before and during anaesthesia were calculated and are shown in Table 1 and Figure 2. In this group, the values were significantly higher during anaesthesia, both for shear wave velocity and Young’s modulus (*p* = 0.002).

**Table 1.** Two-dimensional shear wave elastography liver values for group 1, reported in metre per second (m/s) and kilopascal (kPa) and expressed in median and range. Comparison shear wave velocity and Young’s modulus between awake and anaesthetized dogs

Group 1 (n = 11)	Shear wave velocity (m/s)	Young’s modulus (kPa)
Awake dogs	1.3 (1.22–1.4)	4.9 (4.5–5.8)
Anaesthetized dogs	1.43 (1.33–1.78)	6.05 (5.1–9.4)
	*p* = 0.002	*p* = 0.002

Moreover, we wanted to compare the 2D-SWE values of all awake patients included with those of all dogs subjected to anaesthesia. Therefore, we merged the pre-anaesthesia values of dogs included in group 1 with the values of group 2 (group 1 + 2; n = 30), and the anaesthesia values of dogs included in group 1 with the values of group 3 (group 1 + 3; n = 26).

**Figure 3.**
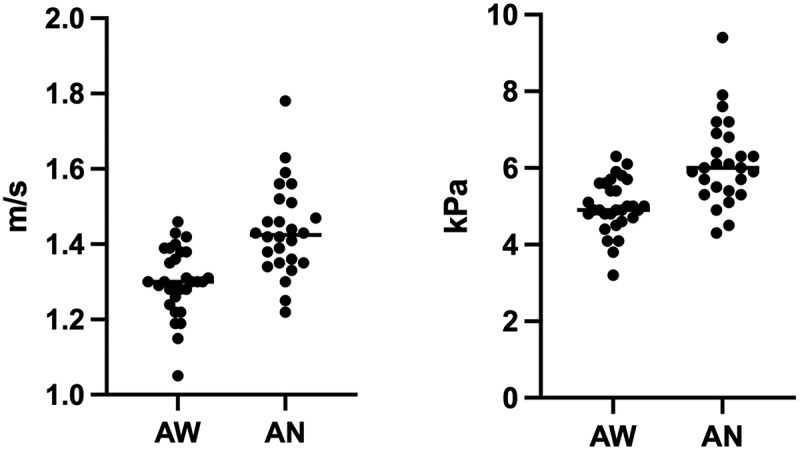
Scatter plots of the shear velocity values in awake dogs (AW) of group 1 + 2 and anaesthetized dogs (AN) of group 1 + 3, expressed in metre per second (m/s) and kilopascal (kPa).

The median 2D-SWE values of group 1 + 2 and group 1 + 3 were calculated and are shown in Table 2 and Figure 3. In these groups too, LS values were significantly higher during anaesthesia, both for shear wave velocity and Young’s modulus (*p* < 0.001).

**Table 2.** Two-dimensional shear wave elastography (2D-SWE) liver values for group 1 + 2 and group 1 + 3, reported in metre per second (m/s) and kilopascal (kPa) and expressed in median and range. Comparison of shear wave velocity and Young’s modulus between awake and anaesthetized dogs

		Shear wave velocity (m/s)	Young’s modulus (kPa)
Group 1 + 2 (n = 30)	Awake dogs	1.3 (1.05–1.46)	4.9 (3.2–6.3)
Group 1 + 3 (n = 26)	Anaesthetized dogs	1.42 (1.22–1.78)	6 (4.3–9.4)
		*p* < 0.001	*p*< 0.001

## 4. Discussion

The aim of our study was to evaluate if our anaesthetic protocol could have an influence on 2D-SWE measurements for assessment of LS in a group of healthy dogs. In 15/26 (57.7%) dogs subjected to anaesthesia, it was not possible to perform the 2D-SWE when they were awake due to movement of the patient and/or tachypnoea. This result showed that the use of anaesthesia could be necessary in many cases to perform liver 2D-SWE in dogs, depending on the cooperation of the animal. This difficulty was observed by also other authors, who identified motion and painting as an important practical limitation in the evaluation of elastography of canine liver [3,8]. In our study, anaesthesia allowed us to overcome this hindrance. Anaesthesia necessity was also observed in one study about source of variability of 2D-SWE of the spleen in healthy dogs[7].

In our study, LS values were significantly higher under anaesthesia and a possible hypothesis is that our anaesthetic protocol had an influence on the hepatic haemodynamics, leading to an increase of 2D-SWE values. In human medicine, hepatic congestion can represent a “confounding factor” for fibrosis staging, determining higher values of SWE [1,2]. The liver has a poorly distensible capsule, and for this reason, hepatic congestion may lead to an increase of LS[12]. In human medicine, an increase of liver SWE values due to hepatic congestion was observed in the course of right-sided heart failure, some congenital heart diseases and valvular diseases[12].

Drugs included in our anaesthetic protocol could have been responsible for multiple and different cardiovascular modifications, thus leading to modifications of liver perfusion [13–17].

Dexmedetomidine is an alpha-2 agonist which causes haemodynamic effects associated with a biphasic blood pressure response, initially characterized by peripheral vasoconstriction, hypertension and bradycardia and after by a reduction in blood pressure [13,18]. A study on 2D-SWE of the spleen in healthy dogs showed increased stiffness of the spleen under anaesthesia, where the drug used was medetomidine[7]. This finding was attributed by the authors to a possible haemodynamic effect of medetomidine on the spleen (modification in blood pressure and vasodilation)[7]. Furthermore, perfusion modifications in the liver caused by dexmedetomidine were also observed by a Contrast-Enhanced-Ultrasound (CEUS) study carried out in dogs[19].

Methadone is a synthetic opioid and can cause disturbances in cardiac conduction following oral and intravenous administration in humans and dogs [14,20,21]; however, in our study, it was administered intramuscularly. Unfortunately, based on the authors’ knowledge, there are no studies available about possible effects of methadone on hepatic perfusion.

Ketamine is a dissociative anaesthetic, which can lead to cardiovascular modifications in humans and dogs such as increase in heart rate and increase in blood pressure [15,22]. An increased spleen size was observed in dogs treated with ketamine, and it was attributed to a possible cardiovascular effect, but it should be considered that these dogs were also treated with medetomidine[23].

Propofol is an intravenous anaesthetic, which can determine a decrease in arterial blood pressure, but with a well-maintained cardiac output in dogs[16]. A concomitant increase of hepatic arterial blood flow was also observed[24]. However, CEUS studies carried out in liver of healthy dogs did not show any alteration in blood flow parameters representing blood volume [25,26].

Finally, isoflurane is an inhalational anaesthetic, which can cause vasodilation and decrease of mean arterial pressure and hepatic resistance in dogs [17,27].

Values obtained in our study for anaesthetized dogs were higher than the ones of awake dogs; however, they are similar to the ones obtained in another study carried out on 8 healthy beagles, performed without anaesthesia (1.51 ± 0.08 m/s and 6.93 ± 0.79 kPa)[4]. This finding supports the hypothesis that our value under anaesthesia could be included in a reference range of normality. Moreover, values obtained in our study for awake dogs appear to be slightly lower than the ones in the study by Tamura et al. Considering that the ultrasound method was the same used for our and Tamura’s study, a possible variability of LS related to breed and/or weight should be considered. Precisely, we included in our study dogs of different breeds with a median body weight of 27.8 kg, while Tamura’s study included only beagles with a body weight between 9.7 and 15 kg. Moreover, our LS values for anaesthetized dogs were lower compared to the median and range described in dogs with clinically relevant hepatic fibrosis (2.04 m/s; range, 1.81–2.26 m/s)[5]; however, it should be considered that LS values variability due to anaesthesia could have an influence in the classification of the degree of hepatic fibrosis. Precisely, a recent study about 2D-SWE for evaluation of human liver fibrosis showed small differences (from 1.6 to 2.8 kPa) between the cut-off of LS values for the fibrosis staging[28].

The principal limits of our study are that we evaluated only one anaesthetic protocol and it was composed of multiple drugs, and therefore, it is not possible to know which drugs or which association of drugs caused the increase of LS. Finally, the 2D-SWE was performed after 40–45 minutes after the premedication and not suddenly after. In clinical practice, if anaesthesia would be used to perform 2D-SWE, the procedure would be probably done immediately after the onset of the anaesthesia effect. On the basis of our study, it is not possible to exclude a possible influence of the time elapsed between premedication/induction of anaesthesia and 2D-SWE measurements, thus producing a disturbing effect on haemodynamics of the liver.

## 5. Conclusion

According to our study, anaesthesia helped us to avoid challenges related to patient movement and respiration to perform 2D-SWE of the liver in healthy dogs. However, it was a source of variability on LS values, and this factor should be considered before performing 2D-SWE under anaesthesia. It is possible that our LS values under anaesthesia could be included in a range of normality for healthy dogs. However, a reference interval of liver 2D-SWE values of healthy dogs and cut-offs to discriminate between pathologic and normal values in dogs are not yet available, and we cannot exclude that our measurements during anaesthesia could mimic a pathological finding or have an influence in the fibrosis staging. Further studies are needed to evaluate the effect of different anaesthetic protocols on 2D-SWE and how they could have an influence on LS values.

## Data Availability

The data set used and analyzed in the current study are available from the first author on reasonable request.
